# Di-μ-oxido-bis­[bis­(diiso­propyl­aceta­midinato)-κ*N*;κ^2^ *N*,*N*′-germanium(IV)]

**DOI:** 10.1107/S1600536813032133

**Published:** 2013-11-30

**Authors:** Ronny Syre, Nancy Frenzel, Cristian G. Hrib, Edmund P. Burte, Peter G. Jones, Frank T. Edelmann

**Affiliations:** a Chemisches Institut, Otto-von-Guericke-Universität Magdeburg, Universitätsplatz 2, D-39106 Magdeburg, Germany; b Institut für Mikro- und Sensorsysteme, Otto-von-Guericke-Universität Magdeburg, Universitätsplatz 2, D-39106 Magdeburg, Germany; c Institut für Anorganische und Analytische Chemie, Technische Universität Braunschweig, Hagenring 30, D-38106 Braunschweig, Germany

## Abstract

The title compound, [Ge_2_(C_8_H_17_N_2_)_4_O_2_], crystallizes with imposed twofold symmetry, which allows the monodentate amidinate ligands to be arranged in a *cisoid* fashion. The independent Ge—O distances within the central Ge_2_O_2_ ring, which is essentially planar (r.m.s. deviation = 0.039 Å), are 1.7797 (8) and 1.8568 (8) Å. The germanium centres adopt a distorted trigonal–bipyramidal geometry, being coordinated by the two O atoms and by one bidentate and one monodentate amidinate ligand (three N atoms). One *N*-isopropyl group is disordered over two positions; these are mutually exclusive because of ‘collisions’ between symmetry-equivalent methyl groups and thus each has 0.5 occupancy.

## Related literature

For comprehensive reviews on metal amidinates and guanidinates, see: Edelmann (2008 ▶, 2013 ▶). For information on germanium precursors for CVD or ALD production of GST thin layers, see: Chen *et al.* (2007 ▶, 2009 ▶, 2010 ▶); Lee *et al.* (2007 ▶). For previous literature on related germanium amidinates, see: Brück *et al.* (2012 ▶); Cabeza *et al.* (2013 ▶); Foley *et al.* (1997 ▶, 2000 ▶); Jones *et al.* (2008 ▶); Jutzi *et al.* (1999 ▶); Karsch *et al.* (1998 ▶); Kühl (2004 ▶); Matioszek *et al.* (2012 ▶); Yeong *et al.* (2012 ▶); Zhang & So (2011 ▶).
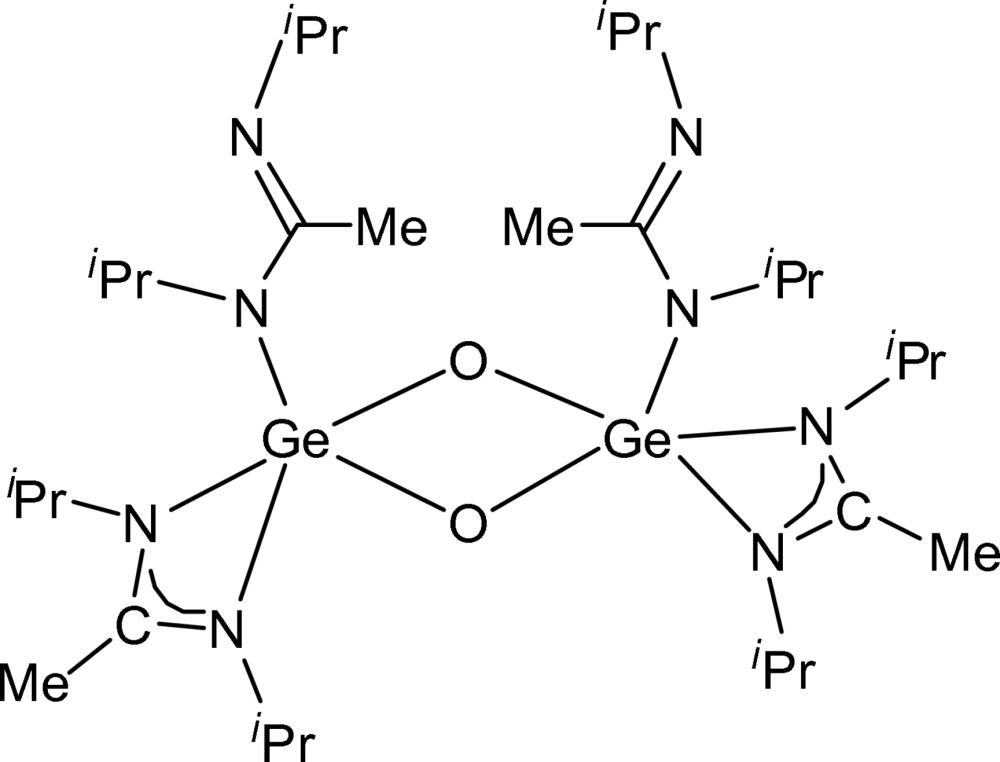


## Experimental

### 

#### Crystal data

[Ge_2_(C_8_H_17_N_2_)_4_O_2_]
*M* *_r_* = 742.12
Monoclinic, 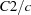

*a* = 20.1934 (2) Å
*b* = 12.7424 (1) Å
*c* = 15.9008 (1) Å
β = 100.038 (1)°
*V* = 4028.84 (6) Å^3^
*Z* = 4
Cu *K*α radiation
μ = 2.11 mm^−1^
*T* = 100 K
0.08 × 0.08 × 0.04 mm

#### Data collection

Oxford Diffraction Xcalibur (Atlas, Nova) diffractometer
Absorption correction: multi-scan (*CrysAlis PRO*; Agilent, 2012 ▶) *T*_min_ = 0.837, *T*_max_ = 1.000
35712 measured reflections
4173 independent reflections
4014 reflections with *I* > 2σ(*I*)
*R*_int_ = 0.021

#### Refinement

*R*[*F* ^2^ > 2σ(*F* ^2^)] = 0.020
*wR*(*F* ^2^) = 0.057
*S* = 1.08
4173 reflections
245 parameters
28 restraints
H-atom parameters constrained
Δρ_max_ = 0.27 e Å^−3^
Δρ_min_ = −0.29 e Å^−3^

### 

Data collection: *CrysAlis PRO* (Agilent, 2012 ▶); cell refinement: *CrysAlis PRO*; data reduction: *CrysAlis PRO*; program(s) used to solve structure: *SHELXS97* (Sheldrick, 2008 ▶); program(s) used to refine structure: *SHELXL97* (Sheldrick, 2008 ▶); molecular graphics: *XP* (Siemens, 1994 ▶); software used to prepare material for publication: *publCIF* (Westrip, 2010 ▶).

## Supplementary Material

Crystal structure: contains datablock(s) I, New_Global_Publ_Block. DOI: 10.1107/S1600536813032133/zl2570sup1.cif

Structure factors: contains datablock(s) I. DOI: 10.1107/S1600536813032133/zl2570Isup2.hkl

Additional supplementary materials:  crystallographic information; 3D view; checkCIF report

## Figures and Tables

**Fig. 1.**
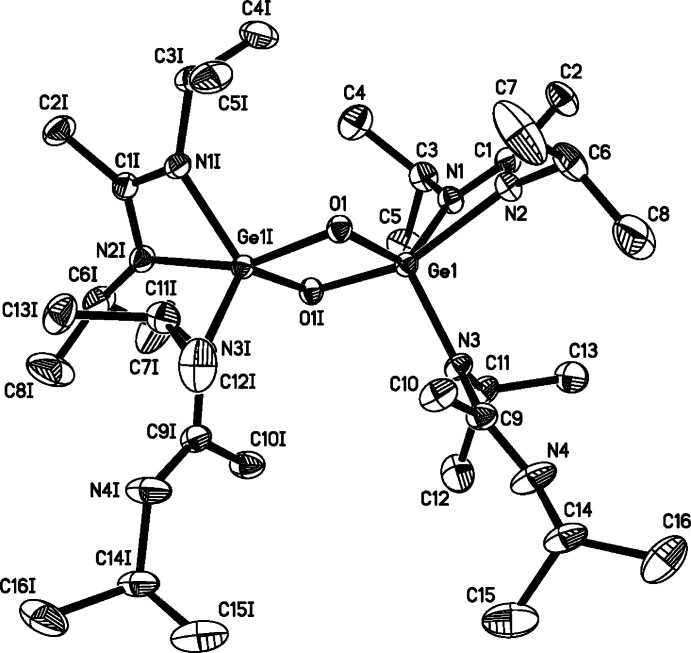
The molecular structure of the title compound in the crystal. Thermal ellipsoids represent 50% probability levels. Only one component of the disordered group N4, C14–16 is shown.

## supplementary crystallographic information

### 1. Comment

Amidinate and guanidinate anions have been widely employed as versatile chelating ligands that form complexes with virtually every metallic element across the Periodic Table (Edelmann 2008, 2013). Among these, germanium compounds comprising amidinate or guanidinate ligands are currently under active investigation as precursors for the deposition of Ge—Sb—Te (= GST) chalcogenide alloys *via* CVD and ALD processes to be used in next generation non-volatile Phase Change Random Access Memory (PRAM) devices (Chen *et al.*, 2007, 2009, 2010; *Lee *et al.**, 2007). The first fully characterized amidinate complexes of germanium were [MeC(NCy)_2_]_2_Ge and [*^t^*BuC(NCy)_2_]_2_Ge, which were both prepared by metathetical reactions between GeCl_2_(dioxane) and the respective lithium amidinates, Li[MeC(NCy)_2_] and Li[*^t^*BuC(NCy)_2_]. The coordination geometry around germanium was found to be distorted tetrahedral, with one of the vertices being occupied by a lone pair of electrons. Both molecules exhibit one chelating and one monodentate ("dangling") amidinate ligand. Mixed amidinato-amido analogues such as [MeC(NCy)_2_]GeN(SiMe_3_)_2_ (*R* = Me, *^t^*Bu) were prepared in a similar manner (Kühl, 2004; Foley *et al.*, 1997, 2000). In contrast, a bis(chelate) structure was found for the closely related germylene [MeC(NPr^i^)_2_]_2_Ge, which was also made from GeCl_2_(dioxane) and two equivalents of the corresponding lithium amidinate (colorless crystals, 81%). The same synthetic approach was used to make bis(amidinato) germanium(IV) dichlorides in high yields (83–95%) (Karsch *et al.*, 1998).

In recent years, amidinate- and guanidinate-stabilized germylenes have become versatile building blocks for novel inorganic ring systems (Cabeza *et al.*, 2013; Matioszek *et al.*, 2012; Yeong *et al.*, 2012), coordination compounds (Jones *et al.*, 2008; Brück *et al.*, 2012), and MOCVD precursors for GST thin-layer deposition (Chen *et al.*, 2007, 2009, 2010). Different reaction products have been isolated from germanium amidinates and chalcogens or chalcogen atom sources. For example, rapid oxidative addition of styrene sulfide or elemental selenium to the germylene derivatives resulted in a series of rare terminal chalcogenido complexes with the formulas [RC(NCy)_2_]_2_Ge═E (*R* = Me, *^t^*Bu; E = S, Se). In a similar manner the amidinato-amido analogues [RC(NCy)_2_][N(SiMe_3_)_2_]Ge═Se (*R* = Me, Bu*^t^*) have been obtained. An X-ray structure determination of the acetamidinate derivative [MeC(NCy)_2_][N(SiMe_3_)_2_]Ge═Se confirmed the presence of a terminal Ge═Se bond (*Foley *et al.**, 1997, 2000). More recently the synthesis and characterization of the amidinate-stabilized bis(germylene) oxide and sulfide LGe—E—GeL (E = O, S; *L* = *^t^*BuC(NAr)_2_, Ar = 2,6-*^i^*Pr_2_C_6_H_3_) have been described. The bis(germylene) oxide was prepared by the reaction of 2 equiv. of LGeCl with Me_3_NO and 2 equiv. of KC_8_ in THF. It has been proposed that the reaction proceeds through an LGe^I^ intermediate, which then reacts with Me_3_NO to form LGe—O—GeL. Similarly, the reaction of two equivalents of LGeCl with elemental sulfur and two equivalents of KC_8_ in THF afforded LGe—S—GeL (Zhang *et al.*, 2011).

To the best of our knowledge, dimeric bis(amidinato)germanium(IV) oxides have not yet been reported in the literature. Such a compound has now been serendipitously obtained in the course of our ongoing investigation of the use of germanium amidinates and guanidinates as new precursors for GST thin-layer deposition. The X-ray crystal structure determination revealed the presence of a *C*_2_-symmetric dimer of the composition [(*µ*-O)Ge{*k*^1^*N*-*N,N'*-MeC(N*^i^*Pr)(=N*^i^*Pr)}{*k*^2^*N,N'*-*N,N'* –MeC(N*^i^*Pr)_2_}]_2_ comprising an almost planar (r.m.s.d. 0.039 Å) central four-membered Ge_2_O_2_ ring (Fig. 1). The independent Ge—O distances are 1.7797 (8) and 1.8568 (8) Å, with an average of 1.8183 Å lying between the Ge—O bond lengths of 1.733 (4) and 1.766 (5) Å in LGe—O—GeL (*L* = *^t^*BuC(NAr)_2_, Ar = 2,6-*^i^*Pr_2_C_6_H_3_) (Zhang *et al.*, 2011) and that in (Mamx)GeO*^i^*Pr (Mamx = methylaminomethyl-*m*-xylyl) of 1.856 (2) Å (Jutzi *et al.*, 1999). In contrast to the germylene precursor [MeC(N*^i^*Pr)_2_]_2_Ge, in which both amidinate ligands are *N,N'*-chelating, each Ge atom in the title compound contains one *N,N'*-chelating and one *k*^1^-coordinated ("dangling") amidinate ligand. The overall *C*_2_ symmetry of the dimeric molecule allows the monodentate amidinate ligands to be arranged in a *cisoid* fashion. The germanium centres adopt a distorted trigonal-bipyramidal geometry, with a bridging oxygen and one N atom of the chelating amidinate arranged in the axial positions (N2—Ge1—O1^i^ 157.00 (3)°). The angle sum around Ge in the equatorial plane (O1, N1, N3) is 358.2 (5)°. The chelating amidinate shows a small bite angle N1—Ge—N2 of 64.80 (4)°, which is typical of this type of heteroallylic ligand (Edelmann 2008, 2013). The C—N bond lengths in the chelating amidinate (C1—N2 1.3038 (15) Å, C1—N1 1.3512 (14) Å) lie approximately between the values for C═N double bonds and C—N(*sp*^2^) single bonds. In the monodentate amidinate ligand, the difference between the formal C═N double bond (N4 disordered: C9—N4 1.281 (12), 1.299 (12) Å) and the C—N(*sp*^2^) single bond (C9—N3 1.3816 (14) Å) is more significant.

### 2. Experimental

The bis(chelated) germylene derivative [MeC(N*^i^*Pr)_2_]_2_Ge was prepared according to the published procedure by treatment of GeCl_2_(dioxane) with two equivalents of (THF)Li[MeC(N*^i^*Pr)_2_] (Karsch *et al.*, 1998). Subsequent recrystallization from *n*-pentane afforded a small amount of well formed, colorless, plate-like single crystals, which were shown by X-ray diffraction to be the title compound. Its formation can only be explained by oxygen contamination during the recrystallization process.

### 3. Refinement

Ordered methyls were refined as idealized rigid groups (C—H 0.98 Å, H—C—H 109.5°) allowed to rotate but not tip; starting positions for the hydrogen sites were taken from a difference synthesis. The methyl group at C2 is rotationally disordered and was refined as above, but with an idealized hexagon of partially occupied alternative hydrogen sites. Other hydrogen atoms were placed in calculated positions and refined using a riding model with C—H_methine_ 1.00 Å; the hydrogen *U* values were fixed at 1.5 × *U*(eq) of the parent atom for methyl H and 1.2 × *U*(eq) of the parent atom for other H.

The *N*-isopropyl group N4, C14–16 is disordered over two positions. These are mutually exclusive because of "collisions" between symmetry-equivalent C16 methyl groups and thus each have occupancies of 0.5. Appropriate similarity restraints were employed to improve stability of refinement. Methyl groups of disordered atoms (C15, C16) were refined using a riding model starting from ideally staggered positions.

### Crystal data

**Table:**

[Ge_2_(C_8_H_17_N_2_)_4_O_2_]	*F*(000) = 1584
*M**_r_* = 742.12	*D*_x_ = 1.224 Mg m^−^^3^
Monoclinic, *C*2/*c*	Cu *K*α radiation, λ = 1.54184 Å
*a* = 20.1934 (2) Å	Cell parameters from 28247 reflections
*b* = 12.7424 (1) Å	θ = 4.1–75.7°
*c* = 15.9008 (1) Å	µ = 2.11 mm^−^^1^
β = 100.038 (1)°	*T* = 100 K
*V* = 4028.84 (6) Å^3^	Plate, colourless
*Z* = 4	0.08 × 0.08 × 0.04 mm

### Data collection

**Table:**

Oxford Diffraction Xcalibur (Atlas, Nova) diffractometer	4173 independent reflections
Radiation source: Nova (Cu) X-ray Source	4014 reflections with *I* > 2σ(*I*)
Mirror monochromator	*R*_int_ = 0.021
Detector resolution: 10.3543 pixels mm^-1^	θ_max_ = 75.9°, θ_min_ = 4.1°
ω scan	*h* = −24→25
Absorption correction: multi-scan (*CrysAlis PRO*; Agilent, 2012)	*k* = −16→15
*T*_min_ = 0.837, *T*_max_ = 1.000	*l* = −19→19
35712 measured reflections	

### Refinement

**Table:**

Refinement on *F*^2^	Secondary atom site location: difference Fourier map
Least-squares matrix: full	Hydrogen site location: inferred from neighbouring sites
*R*[*F*^2^ > 2σ(*F*^2^)] = 0.020	H-atom parameters constrained
*wR*(*F*^2^) = 0.057	*w* = 1/[σ^2^(*F*_o_^2^) + (0.0296*P*)^2^ + 2.8972*P*] where *P* = (*F*_o_^2^ + 2*F*_c_^2^)/3
*S* = 1.08	(Δ/σ)_max_ = 0.002
4173 reflections	Δρ_max_ = 0.27 e Å^−^^3^
245 parameters	Δρ_min_ = −0.29 e Å^−^^3^
28 restraints	Extinction correction: *SHELXL97* (Sheldrick, 2008), Fc^*^=kFc[1+0.001xFc^2^λ^3^/sin(2θ)]^-1/4^
Primary atom site location: structure-invariant direct methods	Extinction coefficient: 0.000087 (13)

### Special details

**Table:**

Geometry. All e.s.d.'s (except the e.s.d. in the dihedral angle between two l.s. planes) are estimated using the full covariance matrix. The cell e.s.d.'s are taken into account individually in the estimation of e.s.d.'s in distances, angles and torsion angles; correlations between e.s.d.'s in cell parameters are only used when they are defined by crystal symmetry. An approximate (isotropic) treatment of cell e.s.d.'s is used for estimating e.s.d.'s involving l.s. planes.
Refinement. Refinement of *F*^2^ against ALL reflections. The weighted *R*-factor *wR* and goodness of fit *S* are based on *F*^2^, conventional *R*-factors *R* are based on *F*, with *F* set to zero for negative *F*^2^. The threshold expression of *F*^2^ > σ(*F*^2^) is used only for calculating *R*-factors(gt) *etc*. and is not relevant to the choice of reflections for refinement. *R*-factors based on *F*^2^ are statistically about twice as large as those based on *F*, and *R*- factors based on ALL data will be even larger.

### Fractional atomic coordinates and isotropic or equivalent isotropic displacement parameters (Å^2^)

**Table:**

	*x*	*y*	*z*	*U*_iso_*/*U*_eq_	Occ. (<1)
Ge1	0.439398 (6)	0.244195 (9)	0.200846 (8)	0.01509 (6)	
O1	0.52408 (4)	0.25038 (5)	0.18371 (5)	0.01763 (17)	
N1	0.37824 (5)	0.35942 (7)	0.19850 (6)	0.01908 (19)	
N2	0.39972 (5)	0.30266 (8)	0.07745 (6)	0.01980 (19)	
N3	0.39391 (5)	0.11656 (7)	0.19497 (6)	0.01919 (19)	
C1	0.36719 (5)	0.37371 (9)	0.11299 (7)	0.0202 (2)	
C2	0.32297 (7)	0.45872 (10)	0.06893 (8)	0.0308 (3)	
H2A	0.3174	0.4490	0.0070	0.046*	0.680 (18)
H2B	0.3437	0.5272	0.0843	0.046*	0.680 (18)
H2C	0.2789	0.4556	0.0867	0.046*	0.680 (18)
H2D	0.3093	0.5055	0.1117	0.046*	0.320 (18)
H2E	0.2830	0.4273	0.0343	0.046*	0.320 (18)
H2F	0.3477	0.4989	0.0320	0.046*	0.320 (18)
C3	0.36233 (6)	0.43549 (9)	0.26152 (7)	0.0231 (2)	
H3	0.3236	0.4791	0.2331	0.028*	
C4	0.42119 (7)	0.50889 (11)	0.29111 (9)	0.0340 (3)	
H4A	0.4595	0.4684	0.3208	0.051*	
H4B	0.4081	0.5615	0.3301	0.051*	
H4C	0.4339	0.5441	0.2415	0.051*	
C5	0.34071 (7)	0.37987 (11)	0.33699 (8)	0.0321 (3)	
H5A	0.3003	0.3380	0.3169	0.048*	
H5B	0.3309	0.4320	0.3784	0.048*	
H5C	0.3770	0.3337	0.3643	0.048*	
C6	0.39731 (7)	0.28663 (11)	−0.01370 (8)	0.0301 (3)	
H6	0.3740	0.3482	−0.0448	0.036*	
C7	0.46850 (10)	0.28230 (19)	−0.03182 (10)	0.0619 (6)	
H7A	0.4911	0.3492	−0.0157	0.093*	
H7B	0.4673	0.2695	−0.0928	0.093*	
H7C	0.4931	0.2254	0.0014	0.093*	
C8	0.35668 (12)	0.18830 (13)	−0.04264 (10)	0.0604 (6)	
H8A	0.3792	0.1269	−0.0135	0.091*	
H8B	0.3532	0.1799	−0.1045	0.091*	
H8C	0.3115	0.1949	−0.0285	0.091*	
C9	0.40646 (6)	0.02753 (9)	0.15071 (7)	0.0216 (2)	
C10	0.47448 (6)	0.02267 (9)	0.12292 (8)	0.0266 (3)	
H10A	0.5042	−0.0244	0.1610	0.040*	
H10B	0.4942	0.0931	0.1255	0.040*	
H10C	0.4690	−0.0039	0.0643	0.040*	
C11	0.33400 (6)	0.12089 (9)	0.23715 (8)	0.0236 (2)	
H11	0.3388	0.1868	0.2718	0.028*	
C12	0.33045 (7)	0.03191 (13)	0.30052 (9)	0.0370 (3)	
H12A	0.3182	−0.0335	0.2692	0.055*	
H12B	0.2965	0.0485	0.3356	0.055*	
H12C	0.3744	0.0236	0.3374	0.055*	
C13	0.26852 (6)	0.13273 (11)	0.17384 (9)	0.0320 (3)	
H13A	0.2720	0.1928	0.1364	0.048*	
H13B	0.2314	0.1442	0.2050	0.048*	
H13C	0.2601	0.0688	0.1394	0.048*	
N4	0.3607 (6)	−0.0436 (7)	0.1284 (5)	0.0219 (13)	0.50
C14	0.3734 (4)	−0.1437 (6)	0.0885 (4)	0.0274 (15)	0.50
H14	0.4104	−0.1343	0.0548	0.033*	0.50
C15	0.3947 (3)	−0.2241 (3)	0.1584 (3)	0.0514 (9)	0.50
H15A	0.4348	−0.1987	0.1969	0.077*	0.50
H15B	0.4051	−0.2907	0.1327	0.077*	0.50
H15C	0.3581	−0.2347	0.1906	0.077*	0.50
C16	0.30970 (18)	−0.1764 (3)	0.0291 (2)	0.0430 (8)	0.50
H16A	0.2969	−0.1219	−0.0142	0.064*	0.50
H16B	0.2734	−0.1860	0.0620	0.064*	0.50
H16C	0.3176	−0.2426	0.0009	0.064*	0.50
N4'	0.3645 (7)	−0.0479 (7)	0.1505 (5)	0.0255 (15)	0.50
C14'	0.3827 (5)	−0.1470 (7)	0.1139 (5)	0.0335 (16)	0.50
H14'	0.4326	−0.1515	0.1188	0.040*	0.50
C15'	0.3495 (3)	−0.1540 (3)	0.0203 (3)	0.0619 (11)	0.50
H15D	0.3689	−0.1008	−0.0126	0.093*	0.50
H15E	0.3011	−0.1419	0.0153	0.093*	0.50
H15F	0.3573	−0.2239	−0.0018	0.093*	0.50
C16'	0.3578 (3)	−0.2357 (3)	0.1644 (3)	0.0570 (12)	0.50
H16D	0.3818	−0.2337	0.2236	0.086*	0.50
H16E	0.3660	−0.3032	0.1386	0.086*	0.50
H16F	0.3094	−0.2273	0.1638	0.086*	0.50

### Atomic displacement parameters (Å^2^)

**Table:**

	*U*^11^	*U*^22^	*U*^33^	*U*^12^	*U*^13^	*U*^23^
Ge1	0.01334 (8)	0.01533 (8)	0.01667 (8)	0.00042 (4)	0.00282 (5)	−0.00043 (4)
O1	0.0150 (4)	0.0206 (4)	0.0175 (4)	−0.0007 (3)	0.0031 (3)	0.0003 (3)
N1	0.0190 (4)	0.0190 (4)	0.0191 (4)	0.0041 (4)	0.0028 (3)	−0.0008 (4)
N2	0.0208 (5)	0.0214 (5)	0.0169 (4)	0.0033 (4)	0.0023 (3)	0.0011 (3)
N3	0.0160 (4)	0.0183 (4)	0.0241 (5)	−0.0020 (3)	0.0058 (3)	−0.0019 (4)
C1	0.0183 (5)	0.0196 (5)	0.0220 (5)	0.0004 (4)	0.0020 (4)	0.0006 (4)
C2	0.0372 (7)	0.0278 (6)	0.0257 (6)	0.0132 (5)	0.0012 (5)	0.0028 (5)
C3	0.0237 (6)	0.0218 (5)	0.0242 (6)	0.0063 (4)	0.0058 (4)	−0.0031 (4)
C4	0.0377 (7)	0.0273 (6)	0.0374 (7)	−0.0013 (5)	0.0074 (6)	−0.0128 (5)
C5	0.0388 (7)	0.0322 (7)	0.0292 (6)	0.0091 (6)	0.0164 (5)	−0.0014 (5)
C6	0.0408 (7)	0.0322 (7)	0.0171 (6)	0.0140 (6)	0.0041 (5)	0.0013 (5)
C7	0.0561 (11)	0.1062 (15)	0.0287 (8)	0.0446 (11)	0.0226 (7)	0.0206 (9)
C8	0.1093 (16)	0.0335 (8)	0.0285 (7)	0.0050 (9)	−0.0156 (9)	−0.0068 (6)
C9	0.0202 (5)	0.0178 (5)	0.0275 (6)	0.0012 (4)	0.0060 (4)	0.0006 (4)
C10	0.0236 (6)	0.0221 (6)	0.0362 (7)	−0.0009 (5)	0.0115 (5)	−0.0049 (5)
C11	0.0190 (5)	0.0241 (6)	0.0295 (6)	−0.0041 (4)	0.0092 (4)	−0.0043 (5)
C12	0.0257 (6)	0.0545 (9)	0.0320 (7)	−0.0064 (6)	0.0087 (5)	0.0118 (6)
C13	0.0184 (6)	0.0322 (7)	0.0458 (8)	0.0016 (5)	0.0067 (5)	0.0079 (6)
N4	0.0254 (19)	0.0206 (16)	0.023 (3)	−0.0030 (12)	0.012 (3)	−0.005 (2)
C14	0.032 (3)	0.0166 (16)	0.037 (4)	−0.0058 (15)	0.015 (3)	−0.012 (2)
C15	0.066 (3)	0.0216 (16)	0.069 (3)	0.0049 (18)	0.021 (2)	−0.0015 (16)
C16	0.0405 (18)	0.0405 (18)	0.0509 (18)	−0.0121 (14)	0.0162 (15)	−0.0264 (14)
N4'	0.034 (2)	0.0169 (16)	0.029 (4)	−0.0040 (12)	0.014 (3)	−0.006 (2)
C14'	0.035 (3)	0.028 (2)	0.040 (4)	−0.0088 (17)	0.013 (3)	−0.016 (2)
C15'	0.072 (3)	0.045 (2)	0.064 (3)	−0.003 (2)	0.002 (2)	−0.0315 (19)
C16'	0.074 (3)	0.0186 (15)	0.088 (3)	−0.0006 (18)	0.041 (3)	−0.0027 (15)

### Geometric parameters (Å, º)

**Table:**

Ge1—O1	1.7797 (8)	C8—H8C	0.9800
Ge1—O1^i^	1.8568 (8)	C9—N4'	1.281 (12)
Ge1—N3	1.8621 (9)	C9—N4	1.299 (12)
Ge1—N1	1.9148 (9)	C9—C10	1.5157 (15)
Ge1—N2	2.1211 (9)	C10—H10A	0.9800
O1—Ge1^i^	1.8568 (8)	C10—H10B	0.9800
N1—C1	1.3512 (14)	C10—H10C	0.9800
N1—C3	1.4694 (14)	C11—C13	1.5234 (17)
N2—C1	1.3038 (15)	C11—C12	1.5268 (18)
N2—C6	1.4560 (14)	C11—H11	1.0000
N3—C9	1.3816 (14)	C12—H12A	0.9800
N3—C11	1.4831 (14)	C12—H12B	0.9800
C1—C2	1.4978 (16)	C12—H12C	0.9800
C2—H2A	0.9800	C13—H13A	0.9800
C2—H2B	0.9800	C13—H13B	0.9800
C2—H2C	0.9800	C13—H13C	0.9800
C2—H2D	0.9800	N4—C14	1.467 (9)
C2—H2E	0.9800	C14—C16	1.516 (7)
C2—H2F	0.9800	C14—C15	1.517 (7)
C3—C4	1.5211 (17)	C14—H14	1.0000
C3—C5	1.5218 (17)	C15—H15A	0.9800
C3—H3	1.0000	C15—H15B	0.9800
C4—H4A	0.9800	C15—H15C	0.9800
C4—H4B	0.9800	C16—H16A	0.9800
C4—H4C	0.9800	C16—H16B	0.9800
C5—H5A	0.9800	C16—H16C	0.9800
C5—H5B	0.9800	N4'—C14'	1.463 (9)
C5—H5C	0.9800	C14'—C16'	1.522 (9)
C6—C7	1.516 (2)	C14'—C15'	1.524 (7)
C6—C8	1.524 (2)	C14'—H14'	1.0000
C6—H6	1.0000	C15'—H15D	0.9800
C7—H7A	0.9800	C15'—H15E	0.9800
C7—H7B	0.9800	C15'—H15F	0.9800
C7—H7C	0.9800	C16'—H16D	0.9800
C8—H8A	0.9800	C16'—H16E	0.9800
C8—H8B	0.9800	C16'—H16F	0.9800
			
O1—Ge1—O1^i^	85.57 (4)	H7A—C7—H7C	109.5
O1—Ge1—N3	120.65 (4)	H7B—C7—H7C	109.5
O1^i^—Ge1—N3	101.19 (4)	C6—C8—H8A	109.5
O1—Ge1—N1	126.64 (4)	C6—C8—H8B	109.5
O1^i^—Ge1—N1	97.49 (4)	H8A—C8—H8B	109.5
N3—Ge1—N1	110.97 (4)	C6—C8—H8C	109.5
O1—Ge1—N2	93.50 (4)	H8A—C8—H8C	109.5
O1^i^—Ge1—N2	157.00 (3)	H8B—C8—H8C	109.5
N3—Ge1—N2	99.02 (4)	N4'—C9—N3	116.0 (5)
N1—Ge1—N2	64.80 (4)	N4—C9—N3	121.8 (5)
Ge1—O1—Ge1^i^	94.21 (4)	N4'—C9—C10	126.9 (5)
C1—N1—C3	125.44 (9)	N4—C9—C10	122.0 (5)
C1—N1—Ge1	96.65 (7)	N3—C9—C10	115.98 (10)
C3—N1—Ge1	135.19 (7)	C9—C10—H10A	109.5
C1—N2—C6	126.65 (10)	C9—C10—H10B	109.5
C1—N2—Ge1	88.89 (7)	H10A—C10—H10B	109.5
C6—N2—Ge1	144.41 (8)	C9—C10—H10C	109.5
C9—N3—C11	119.83 (9)	H10A—C10—H10C	109.5
C9—N3—Ge1	127.60 (8)	H10B—C10—H10C	109.5
C11—N3—Ge1	112.27 (7)	N3—C11—C13	112.79 (10)
N2—C1—N1	109.59 (10)	N3—C11—C12	114.00 (10)
N2—C1—C2	127.14 (11)	C13—C11—C12	112.00 (10)
N1—C1—C2	123.27 (10)	N3—C11—H11	105.7
N2—C1—Ge1	59.23 (6)	C13—C11—H11	105.7
N1—C1—Ge1	50.41 (5)	C12—C11—H11	105.7
C2—C1—Ge1	173.51 (9)	C11—C12—H12A	109.5
C1—C2—H2A	109.5	C11—C12—H12B	109.5
C1—C2—H2B	109.5	H12A—C12—H12B	109.5
H2A—C2—H2B	109.5	C11—C12—H12C	109.5
C1—C2—H2C	109.5	H12A—C12—H12C	109.5
H2A—C2—H2C	109.5	H12B—C12—H12C	109.5
H2B—C2—H2C	109.5	C11—C13—H13A	109.5
C1—C2—H2D	109.5	C11—C13—H13B	109.5
H2A—C2—H2D	141.1	H13A—C13—H13B	109.5
H2B—C2—H2D	56.3	C11—C13—H13C	109.5
H2C—C2—H2D	56.3	H13A—C13—H13C	109.5
C1—C2—H2E	109.5	H13B—C13—H13C	109.5
H2A—C2—H2E	56.3	C9—N4—C14	123.7 (9)
H2B—C2—H2E	141.1	N4—C14—C16	108.3 (7)
H2C—C2—H2E	56.3	N4—C14—C15	108.6 (6)
H2D—C2—H2E	109.5	C16—C14—C15	111.9 (6)
C1—C2—H2F	109.5	N4—C14—H14	109.3
H2A—C2—H2F	56.3	C16—C14—H14	109.3
H2B—C2—H2F	56.3	C15—C14—H14	109.3
H2C—C2—H2F	141.1	C14—C15—H15A	109.5
H2D—C2—H2F	109.5	C14—C15—H15B	109.5
H2E—C2—H2F	109.5	H15A—C15—H15B	109.5
N1—C3—C4	111.39 (10)	C14—C15—H15C	109.5
N1—C3—C5	110.94 (10)	H15A—C15—H15C	109.5
C4—C3—C5	111.01 (11)	H15B—C15—H15C	109.5
N1—C3—H3	107.8	C14—C16—H16A	109.5
C4—C3—H3	107.8	C14—C16—H16B	109.5
C5—C3—H3	107.8	H16A—C16—H16B	109.5
C3—C4—H4A	109.5	C14—C16—H16C	109.5
C3—C4—H4B	109.5	H16A—C16—H16C	109.5
H4A—C4—H4B	109.5	H16B—C16—H16C	109.5
C3—C4—H4C	109.5	C9—N4'—C14'	115.9 (10)
H4A—C4—H4C	109.5	N4'—C14'—C16'	107.6 (7)
H4B—C4—H4C	109.5	N4'—C14'—C15'	109.9 (6)
C3—C5—H5A	109.5	C16'—C14'—C15'	110.0 (6)
C3—C5—H5B	109.5	N4'—C14'—H14'	109.8
H5A—C5—H5B	109.5	C16'—C14'—H14'	109.8
C3—C5—H5C	109.5	C15'—C14'—H14'	109.8
H5A—C5—H5C	109.5	C14'—C15'—H15D	109.5
H5B—C5—H5C	109.5	C14'—C15'—H15E	109.5
N2—C6—C7	109.03 (11)	H15D—C15'—H15E	109.5
N2—C6—C8	109.80 (12)	C14'—C15'—H15F	109.5
C7—C6—C8	113.02 (15)	H15D—C15'—H15F	109.5
N2—C6—H6	108.3	H15E—C15'—H15F	109.5
C7—C6—H6	108.3	C14'—C16'—H16D	109.5
C8—C6—H6	108.3	C14'—C16'—H16E	109.5
C6—C7—H7A	109.5	H16D—C16'—H16E	109.5
C6—C7—H7B	109.5	C14'—C16'—H16F	109.5
H7A—C7—H7B	109.5	H16D—C16'—H16F	109.5
C6—C7—H7C	109.5	H16E—C16'—H16F	109.5
			
O1^i^—Ge1—O1—Ge1^i^	4.88 (4)	C3—N1—C1—Ge1	163.67 (13)
N3—Ge1—O1—Ge1^i^	−95.52 (4)	O1—Ge1—C1—N2	58.62 (7)
N1—Ge1—O1—Ge1^i^	100.93 (4)	O1^i^—Ge1—C1—N2	161.01 (6)
N2—Ge1—O1—Ge1^i^	161.84 (3)	N3—Ge1—C1—N2	−76.93 (7)
O1—Ge1—N1—C1	72.88 (8)	N1—Ge1—C1—N2	−177.09 (11)
O1^i^—Ge1—N1—C1	162.93 (7)	O1—Ge1—C1—N1	−124.28 (7)
N3—Ge1—N1—C1	−92.00 (7)	O1^i^—Ge1—C1—N1	−21.89 (9)
N2—Ge1—N1—C1	−1.70 (6)	N3—Ge1—C1—N1	100.16 (7)
O1—Ge1—N1—C3	−88.15 (11)	N2—Ge1—C1—N1	177.09 (11)
O1^i^—Ge1—N1—C3	1.89 (11)	C1—N1—C3—C4	−89.99 (14)
N3—Ge1—N1—C3	106.97 (11)	Ge1—N1—C3—C4	66.67 (14)
N2—Ge1—N1—C3	−162.73 (12)	C1—N1—C3—C5	145.80 (11)
O1—Ge1—N2—C1	−127.45 (7)	Ge1—N1—C3—C5	−57.54 (14)
O1^i^—Ge1—N2—C1	−40.55 (12)	C1—N2—C6—C7	129.19 (15)
N3—Ge1—N2—C1	110.76 (7)	Ge1—N2—C6—C7	−54.4 (2)
N1—Ge1—N2—C1	1.75 (7)	C1—N2—C6—C8	−106.49 (15)
O1—Ge1—N2—C6	55.45 (15)	Ge1—N2—C6—C8	69.89 (19)
O1^i^—Ge1—N2—C6	142.36 (14)	C11—N3—C9—N4'	−1.0 (5)
N3—Ge1—N2—C6	−66.34 (15)	Ge1—N3—C9—N4'	−174.2 (5)
N1—Ge1—N2—C6	−175.35 (16)	C11—N3—C9—N4	15.5 (5)
O1—Ge1—N3—C9	−24.75 (11)	Ge1—N3—C9—N4	−157.6 (4)
O1^i^—Ge1—N3—C9	−116.23 (10)	C11—N3—C9—C10	−169.85 (10)
N1—Ge1—N3—C9	141.17 (9)	Ge1—N3—C9—C10	17.01 (15)
N2—Ge1—N3—C9	74.80 (10)	C9—N3—C11—C13	−73.08 (14)
O1—Ge1—N3—C11	161.68 (7)	Ge1—N3—C11—C13	101.05 (10)
O1^i^—Ge1—N3—C11	70.20 (8)	C9—N3—C11—C12	56.10 (15)
N1—Ge1—N3—C11	−32.40 (9)	Ge1—N3—C11—C12	−129.76 (9)
N2—Ge1—N3—C11	−98.78 (8)	N4'—C9—N4—C14	−102 (4)
C6—N2—C1—N1	175.52 (11)	N3—C9—N4—C14	−174.1 (5)
Ge1—N2—C1—N1	−2.38 (9)	C10—C9—N4—C14	11.6 (9)
C6—N2—C1—C2	−3.6 (2)	C9—N4—C14—C16	−148.6 (7)
Ge1—N2—C1—C2	178.47 (12)	C9—N4—C14—C15	89.7 (9)
C6—N2—C1—Ge1	177.89 (13)	N4—C9—N4'—C14'	72 (3)
C3—N1—C1—N2	166.32 (10)	N3—C9—N4'—C14'	−171.9 (5)
Ge1—N1—C1—N2	2.65 (10)	C10—C9—N4'—C14'	−4.5 (9)
C3—N1—C1—C2	−14.48 (18)	C9—N4'—C14'—C16'	145.1 (7)
Ge1—N1—C1—C2	−178.16 (10)	C9—N4'—C14'—C15'	−95.1 (9)

Symmetry code: (i) −*x*+1, *y*, −*z*+1/2.
